# Inverted Perceptual Judgment of Nociceptive Stimuli at Threshold Level following Inconsistent Cues

**DOI:** 10.1371/journal.pone.0132069

**Published:** 2015-07-06

**Authors:** Carmen Walter, Violeta Dimova, Julia Bu, Michael J. Parnham, Bruno G. Oertel, Jörn Lötsch

**Affiliations:** 1 Institute of Clinical Pharmacology, Goethe—University, Theodor-Stern-Kai 7, 60590, Frankfurt am Main, Germany; 2 Department of Anesthesiology, Intensive Care Medicine and Pain Therapy, University Hospital Frankfurt, Theodor-Stern-Kai 7, 60590, Frankfurt am Main, Germany; 3 Fraunhofer Institute for Molecular Biology and Applied Ecology IME, Project Group Translational Medicine and Pharmacology TMP, Theodor-Stern-Kai 7, 60596, Frankfurt am Main, Germany; Central Institute of Mental Health, GERMANY

## Abstract

**Objective:**

The perception of pain is susceptible to modulation by psychological and contextual factors. It has been shown that subjects judge noxious stimuli as more painful in a respective suggestive context, which disappears when the modifying context is resolved. However, a context in which subjects judge the painfulness of a nociceptive stimulus in exactly the opposite direction to that of the cues has never been shown so far.

**Methods:**

Nociceptive stimuli (300 ms intranasal gaseous CO_2_) at the individual pain threshold level were applied after a visual cue announcing the stimulus as either “no pain”, merely a “stimulus”, or “pain”. Among the stimuli at threshold level, other CO_2_ stimuli that were clearly below or above pain threshold were randomly interspersed. These were announced beforehand in 12 subjects randomly with correct or incorrect cues, i.e., clearly painful or clearly non-painful stimuli were announced equally often as not painful or painful. By contrast, in a subsequent group of another 12 subjects, the stimuli were always announced correctly with respect to the evoked pain.

**Results:**

The random and often incorrect announcement of stimuli clearly below or above pain threshold caused the subjects to rate the stimuli at pain-threshold level in the opposite direction of the cue, i.e., when the stimuli were announced as “pain” significantly more often than as non-painful and vice versa (p < 10^-4^). By contrast, in the absence of incongruence between announcement and perception of the far-from-threshold stimuli, stimuli at pain threshold were rated in the cued direction.

**Conclusions:**

The present study revealed the induction of associations incongruent with a given message in the perception of pain. We created a context of unreliable cues whereby subjects perceived the stimulus opposite to that suggested by a prior cue, i.e., potentially nociceptive stimuli at pain threshold level that were announced as painful were judged as non-painful and vice versa. These findings are consistent with reported data on the effects of distrust on non-painful cognitive responses.

## Introduction

Pain and its relief are known to be susceptible to psychological and contextual factors. Arousal [1,2], expectations regarding the painfulness of stimuli [3–7], attentional bias toward nociceptive stimuli [8], the aversive effects of pain stimuli or the subject’s motivation [9,10], need for analgesic relief [11] or attitudes towards analgesic treatments [5] have all been identified as potential modulators of the individual perception of pain following acute nociceptive input.

Several reports have shown that the perception of nociceptive stimuli is susceptible to cues (for a short overview, see Table 1). The effects have mostly been investigated with respect to an influence of cue-based expectations on clear noxious stimuli (for a review, see [3]). One consistent observation of available studies is that the effects of expectations created prior to stimulus presentation depended on the valence of presented material [12]. The effects of cues on pain perception were also mostly shown to go in the primed direction, e.g. cues suggesting painfulness of upcoming nociceptive stimuli lead to the perception of these stimuli as more painful. For example, pain ratings of noxious electrical stimuli increased following pain-related and negative affective primes as compared to neutral cues as assessed in a semantic priming paradigm [13]. Furthermore, preceding visual cues that denoted either high or low intensity of imminent stimulus enhanced or reduced the perceived pain intensity, accordingly [4]. A clinical example in this context is the enhancement of placebo analgesia in response to subjectively perceived drug side effects [14].

**Table 1:** Summary of studies demonstrating that different expectations regarding imminent nociceptive stimuli may alter pain perception in different experimental contexts. While the study examples showed that the manipulation of perception was dependent on the stimulus context, no study has yet shown that the perception of nociceptive stimuli at threshold can be changed to the opposite of the respective cue.

Reference	Pain stimulus	Stimulus intensity (relative to pain threshold)			Cues	Paradigm	Results (psychophysics)
		Below	Near	Above			
[4]	Contact heat	-	-	X (2 intensities)	"High Temperature", "Low Temperature"	Sets of 20 high or low temperature stimuli each paired with high and low expectation cues, respectively	Stimuli were rated as more painful when expected as such.
[54]	Transcutaneous CO_2_ laser heat	X	-	X	“Uncertain" (pain stimuli might occur) and "certain" (only non-painful stimuli occur)	Sets of 40 stimuli applied in "uncertain context" (painful and non-painful stimuli), 20 stimuli applied in "certain context" (only non-painful stimuli)	Painful stimuli were rated as painful, non-painful stimuli as non-painful; perception of non-painful stimuli under different conditions did not differ between contexts.
[53]	Contact heat (ultra-brief, 250 ms); Five intensities ranging from no pain to maximum pain	X	X	X	Picture of VAS with marks corresponding to ratings of eight fictive people with varying uncertainty	50 stimuli preceded by vicarious information	Uncertainty regarding pain intensity increased pain perception.
[15]	Transcutaneous Laser heat	-	X	-	Picture of site of application of next stimulus	120 stimuli preceded cues suggesting low or high threat condition ("approved" or "approved with reservation”)	More stimuli rated as painful under high threat condition
[10]	Contact cold (-25°C, 500 ms)	-	-	X	Verbal instruction regarding temperature of metal bar (“very hot” or “very cold”)	Metal bar applied, amongst other objects	After prior announcement of hot bar it was rated as more painful
[6]	Contact heat	X	-	X	Red letters “Get ready”	12 heat pain stimuli, half of them preceded by cue; 4 warm stimuli, three of them preceded by cue	Cued painful stimuli were rated as more painful than uncued stimuli
[58]	Contact heat	-	-	X	“High”, “Low”	18 stimuli (6 low pain, 12 high pain), low stimuli were always announced as low; 6 of the high stimuli announced as high, while 6 were announced as low	Stimuli were rated as less painful when expected as such
[65]	Contact heat	-	-	x	Auditory cues (500 and 100 Hz) announcing low and high painful stimulation	High and low pain stimuli announced correctly while medium pain stimuli preceded by cues for low and high pain	Medium painful stimuli rated as more painful when announced as high pain stimuli
[66]	Contact heat	x	-	x	Red, green and blue light announcing warm, painful and resting condition	During signaled rest period pain stimuli was applied and during signaled stimulation no stimulus was applied	No changes in perceived pain intensity or unpleasantness during experiment
[67]	Contact heat	x	-	x	Arrows pointing up or down indicating non-painful and intense painful stimuli, resp.	48 moderate pain stimuli in two contexts representing either worst (instead of no pain) or best (instead of intense pain) outcome	Reduced pain intensity for relative relief context
[20]	Transcutaneous laser heat	x	-	x	“Low”, “Medium”, “High”, “Unknown”	120 stimuli were announced correctly in “certain anticipation” condition, 120 stimuli were applied in “uncertain anticipation” condition	Stimuli applied in “certain anticipation” condition were rated as more painful
[68]	Contact heat	-	-	x	Longer expectation intervals indicated more intense stimulus temperature	30 stimuli applied, 6 were falsely signaled	Expectations of decreased pain reduce pain intensity perception
[13]	Noxious electrical stimuli (20 ms)	-	-	x	Pain-related, negative, positive and neutral adjectives	Semantic priming, total of 230 trials	Elevated pain ratings following pain-related and negative primes vs. neutral cues
[69]	YAG Laser stimuli (1.4 ms, 1.8 μm)	-	-	x	Pain-related affective, pain-related somatosensory and neutral adjectives	Affective priming, 2 experimental blocks, total of 150 trials	no effects of cue category on pain perception

However, only recently a study focus was set at how cue-based expectations influence the perception of noxious stimuli at pain threshold level. First studies showed that nociceptive stimuli at threshold level were more likely to be judged as painful if applied under threat condition [15] whereas cues signaling pain relief mitigated the subsequent pain experience [16]. These studies suggested that even at threshold level noxious stimuli can be perceived as aversive or not and judged accordingly. Moreover, expectation effects on pain ratings further depend on the anticipation of upcoming events created by contextual factors and previous pain experiences [17]. Pain perception was exacerbated in particular when noxious stimuli are applied in an uncertain context rather than in a predictable and stable environment [18,19]. For example, when nociceptive laser heat stimuli were always congruently visually cued to their intensity (“low”, “medium” or “high”), subjects reported lower pain ratings for the low-intensity stimuli [20]. In an uncertain condition where stimuli of different intensities were cued as “unknown”, a similar effect was observed for the high intensity nociceptive stimuli that were rated as more painful [20]. It seems that especially in an uncertain context subjects perceive nociceptive stimuli as more aversive.

However, in all these experiments the effect was always observed in the direction defined by the cues. In other contexts than pain, it has been shown that subjects automatically activate cue-incongruent cognition in a learned context of distrust [21]. Thus, the effect became directed oppositely to the direction defined by the cues. Even when the distrust was unrelated to the message, the cognitive system reacted to distrust by automatically inducing consideration of incongruent associations [21]. On this background, we hypothesized that cue-incongruent associations can also be observed with pain sensations when a condition is created where subjects cannot rely on the pre-announcement of stimuli and moreover, the announcement is often not only unspecific but even contrary to the subsequent perception. Specifically, when creating a context in which stimuli clearly above or below the pain threshold were occasionally incongruently cued, this would influence the judgments of nociceptive stimuli at the pain threshold level in a way that a tendency toward cue-incongruent judgment of these stimuli will be induced.

## Methods

### Subjects and study design

The study followed the Declaration of Helsinki and was approved by the Ethics Committee of the Goethe-University Frankfurt am Main, Germany. Informed written consent from each participating subject had been obtained. Inclusion criteria were age between 18 and 50 years and no relevant current medical history. The subjects’ actual health was ascertained by medical history and physical examination including vital signs. Exclusion criteria were a current clinical condition affecting pain, any other actual diseases and drug intake within a week, except for oral contraceptives. Alcohol was prohibited for 24 h before the actual experiments.

The perception of nociceptive stimuli at threshold level, in a context of cues regarding the imminent sensation of pain, was investigated in 12 healthy volunteers (mean age ± standard deviation: 23.9 ± 1.4 years, sex: 6 men, 6 women). This case number was considered to provide sufficient statistical power based on group comparisons in fMRI assessments [22,23] and was applied (i) because the study was exploratory for such assessments and (ii) numerical data on which a precise case number calculation for the present task could be based was lacking; a post-hoc calculation of sample size was avoided as this has been discouraged [24]. However, to interpret the observations, a second group of 12 subjects was subsequently enrolled (12 women, age 26.5 ± 2.7 years, two-sample t-test [25]: t(22) = -2.912, p < 0.05), in whom a different stimulus paradigm was applied (see below).

### Induction of experimental pain

Short stinging pain was induced by 300 ms-long pulses of gaseous CO_2_ delivered to the nasal mucosa via a Teflon tube with an outer diameter of 4 mm [26,27]. Concomitant alteration of mechanical or thermal conditions at the mucosa was avoided by embedding the CO_2_ pulses in a constantly flowing air stream (8 l/min) with controlled temperature and humidity (36.5°C, 80% relative humidity). A special device (Olfactometer OM/2, Burghart Instruments, Wedel, Germany) precisely controlled the concentration and duration of the rectangular (rise time approximately 50 ms) CO_2_ stimuli [27]. Applied to the nasal mucosa, where CO_2_ is converted into bicarbonate and protons by the enzyme carboanhydrase [28], it evokes a short stinging pain sensation [29] due to excitation of trigeminal nociceptors [26,27] via activation of ion channels such as TRPV1 [30] and TRPA1 [31]. The CO_2_ stimuli activate predominantly trigeminal A_δ_-fibers, with co-activation of C-fibers [32]. Their nociceptive specificity is supported by the demonstration, in a magneto-encephalography experiment, that they evoke cortical potentials generated in the pain-relevant secondary somatosensory area [33]. The pain model has been successfully used in pain research for at least 30 years [27,33]; it can therefore be regarded as being well established [34–36] including in fMRI assessments [37,38]. Random interstimulus intervals of 26.6 ± 2.6 s were used to keep habituation and adaptation to a minimum [39].

### Stimulation paradigm and pain ratings

#### Pain threshold calibration

Each experimental session was preceded by a calibration of the individual pain threshold to the CO_2_ stimuli as implemented in previous studies [4,15]. Using a staircase paradigm, 16 stimuli of different intensities were applied, chosen in random succession of concentrations of 30, 32, 34, 36, 38, 40, 42, 44, 46, 48, 50, 52, 54, 56, 58, 60% v/v CO_2_, with each concentration applied once [29,40]. The threshold determination followed the previously established protocol [41] and all subjects received the same random succession of the CO_2_ stimuli during the pain threshold calibration. The procedure took approximately 8 min. The arithmetic mean of the highest CO_2_ concentration that was judged as not painful and the lowest CO_2_ concentration that was judged as painful was used as the pain threshold. Thresholds were observed at CO_2_ concentrations of 51.6 ± 2.1% v/v in the first group (group 1) and at 51.4 ± 4.3% v/v in the subsequently enrolled second group (group 2). Thus, they were nearly identical between groups (unpaired *t* test: t(22) = -0121, p = 0.9) and corresponded well, with respect to the CO_2_ concentration, to previous findings [29,41]. The success of pain threshold calibration was verified as suggested previously [15], i.e., by establishing a lack of significant difference between the number of stimuli at pain threshold level that were rated as painful or not. This was found to be the case based on non-significant differences (paired t tests: t(11) = -1.77, p = 0.103 and t(11) = 0.853), p = 0.172 for the first and second group of subjects, respectively). Threshold determinations were not an inherent part of the experimental sessions, which were performed as follows.

#### Assessment of perceptual judgments of nociceptive stimuli

The main experiment consisted of the application of 15 physically identical stimuli at a single CO_2_ concentration corresponding to the individual pain threshold level, determined as described above. Stimuli either clearly below (25% v/v CO_2_) or clearly above (75% v/v CO_2_) the pain threshold were applied in a randomly interspersed manner. Subjects were unaware that they received only three different stimulus intensities.

Each stimulus was preceded by a visual cue displayed on a computer screen consisting of either the message “no pain” (yellow font on green background; pain-free condition [42,43]), “stimulus”(black font on light grey background; neutral tactile condition [44]), or “pain” (black font on red background; painful condition [4,42,43]), a paradigm analogously used to elicit placebo and nocebo effects (i.e., decreasing or increasing symptoms) on itch [45]. All colors were adjusted to have the same brightness [46]. The cues were shown for 5.2 ± 0.7 s immediately before stimulus onset and disappeared on stimulus application. Subjects were informed that each stimulus would be announced beforehand. At 3.2 ± 1.4 s after stimulus delivery, subjects were visually prompted to indicate, by pressing computer mouse buttons, whether or not they had perceived the stimulus as painful. Subjects had been instructed to rate according to their perception rather than to the visual cue as “no pain” or “pain”, i.e., a yes/no paradigm. Six seconds later, they rated how sure they were about their decision on a visual analog scale (VAS), ranging from 0, “very uncertain” to 100, “very certain”. Stimulus presentation and response recordings were implemented using the presentation software (Neurobehavioral Systems, Berkeley, CA, USA).

**Fig 1:**
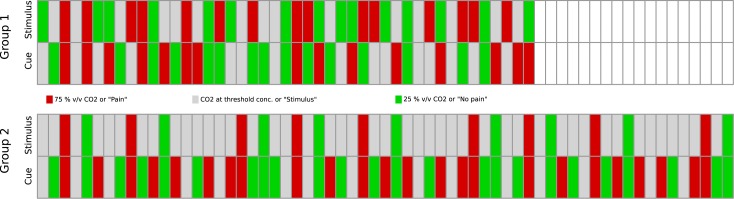
Graphical scheme of the experimental settings applied in either group 1 (top bars) or group 2 (bottom bars) subjects. The paradigm applied to either group is shown in each two lines of differently colored small bars of which each denotes a CO_2_ stimulus and the associated cues. Specifically, in the upper lines, the CO_2_ stimuli are shown in their chronological succession differently colored for 25% v/v CO_2_ stimuli (green), CO_2_ concentration at the individual pain threshold level (grey) and 75% v/v CO_2_ (red). Below each stimulus, in the second line the cues given before stimulus application are denoted as “No pain” (green), “Stimulus”, i.e., a neutral cue (grey) and “Pain” (red). In the first group, the far-from-thresholds stimuli were cued randomly, i.e., green and red bars in lines 1 and 2 are mixed, whereas in the second group, the 25 and 75% stimuli were always preceded by correct cues, which can be seen by the consistent agreement between green or red bars in lines 1 and 2. The inequality of the experimental settings between the groups is commented on in the discussion of this paper.

The cues were given randomly, i.e., both the 25% v/v and the 75% v/v CO_2_ stimuli were randomly announced as “no pain”, neutral (“stimulus”) or “pain” (n = 5 per condition; group 1; for details, see Fig 1), meaning that several of the 75% CO_2_ stimuli were announced as non-painful, despite their clear painfulness, and several of the 25% CO_2_ stimuli were announced as painful, although the evoked pricking sensations were clearly below the pain threshold. This paradigm served to create a context of questioning the cues thus arousing mistrust. The details of the stimulation and cue paradigm are shown in Fig 1 (top). To interpret the results, a second group (group 2) was assessed. In this group, the 25 and 75% CO_2_ stimuli were always announced correctly, i.e., the 25% v/v stimuli were announced as “no pain” and the 75% v/v stimuli as “pain”. This paradigm excluded the application of the neutral cue for the far from pain thresholds stimuli. Thus, the total number of stimuli for group 1 was 45 stimuli (15 threshold stimuli and 15 far-below and far-above threshold stimuli, respectively), whereas subjects of group 2 received 63 stimuli (45 threshold stimuli and nine far-below and far-above threshold stimuli, respectively). For this reason and also due to the sequential performance of the study, the stimulation paradigms differed between groups (Fig 1), which finally posed some problems in the interpretation of the causes underlying the present observations that will be addressed in the discussion to this paper. The main experiments lasted 23 min in the first and 30 min in the second group.

### Statistics

To analyze whether presenting cues in different contexts affected the judgment of whether stimuli at pain threshold were painful or not, the percentages of stimuli at pain threshold level rated as painful were submitted to analysis of variance for repeated measures (rm-ANOVA), with “cue” (“no pain”, “stimulus” or “pain”; degrees of freedom, df = 2) as the within-subject factor and “group” (df = 1) as the between-subject factor. In addition, VAS ratings of the subjects’ certainty about their judgments of the stimuli at pain threshold level were analyzed analogously, however, with the additional within-subjects factor “pain” (yes/no, df = 2) to distinguish between stimuli ratings as either painful or not. Effect sizes for the rm-ANOVA factors were obtained as partial eta square, $\eta_{p}^{2}$_,_ describing the proportion of total variance in the ratings explained by the respective factor [47] as implemented in the SPSS software package (version 22 for Linux, IBM SPSS Statistics, Chicago, USA). By contrast, the ratings for the far-from-threshold stimuli at 25 or 75% v/v CO_2_ were analyzed by means of one-sample t-tests [25], testing the null hypothesis that the population mean is equal to a specified value, against the expected perception of a stimulus as being painful in 0 or 100% of the 25 or 75% v/v CO_2_ stimuli, respectively. The α level was set at 0.05 and corrected for multiple testing according to Bonferroni [48]. Data are reported as arithmetic means ± standard deviation unless indicated otherwise.

Subsequently, to obtain a statistical basis for a more precise interpretation of the ANOVA results, the judgment of stimuli at threshold level as being either painful or not, in differing cue contexts, was further approached by mathematical modeling, as previously applied in a pain context [49]. This analysis established whether changing the context not only reversed the effects of suggestive cues to zero, but whether they clearly inverted them to the opposites. Specifically, the percentages of stimuli at pain threshold level rated as painful were associated with the three different cues using a linear model of $PercentRatedAsPain=Slope\cdot \theta_{1}^{2-Group}\cdot \theta_{2}^{Sex}\cdot Cue+Y\_Intersection\cdot \theta_{3}^{2-Group}\cdot \theta_{4}^{Sex}$, where *Cue* = [–1,0,1] stands for “no pain”, “stimulus” (neutral cue) or “pain”, respectively; *Group* = [1,2] denotes either group 1 or group 2; and *Sex* = [0,1] denotes women or men, respectively. Thus, the subjects’ group and gender were included in this model as covariates as they might have affected the rating as painful of stimuli at pain threshold level. Fitting this model to the individual ratings of stimuli at pain threshold level as painful was done using non-linear mixed effects modeling with NONMEM 7.3 (Icon, Dublin, Ireland [50]) on an Intel Xeon computer running on Ubuntu Linux 14.04 64-bit. This estimated the structural parameters of the model together with their interindividual variance in one single step. The interindividual variability of the model parameters was modeled on the basis of being normally distributed. For instance, the slope of the linear relationship for an individual *i* is given as *Slope*_*i*_ = *Slope*_*TV*_ + η_*i*_, in which *Slope*_*TV*_ is the typical value of the slope in the respective subject group and *η* is a parameter with a mean of zero and a variance of *ω* ^2^ [for details, see [50]] describing the interindividual variance of the slope. During the fitting process, interindividual variances in model parameters and covariates were introduced into the model in a stepwise fashion. Whether or not a specific variance or covariate remained part of the final model was established based on goodness-of-fit assessments. In this regard, the main indicator was the change in minus twofold the log likelihood (Δ_-2LL_) using the *χ* ^*2*^ approximation for which the number of degrees of freedom was equal to the difference in the number of terms between two models. Thus, the full model included an additional term and the reduced model involved the fixing of the respective term to a neutral value, i.e., 1 for factors and exponents and zero for summands [α-level 0.05; for further details of the fitting process see [50]]. The observed percentages of rating as being painful of stimuli at pain threshold level differed from model predictions by the residual error ε, which was modeled using an additive error model of *PercentRatedAsPain*_*Observed*_ = *PercentRatedAsPain*_*Predicted*_ + *ϵ* in which *ϵ* is a parameter with a mean of zero and a variance of σ^2^ that describes an additive error. Calculations were performed using “first order conditional estimation” and “*η-ε* interaction”. Confidence intervals (95%) of parameter values were calculated from 1,000 runs of the final model with data sets that were obtained by Bootstrap resampling [51] from the original data set [52], using PDxPop (version 5.10, Icon, Dublin, Ireland) for NONMEM. The 95% confidence intervals of the parameter values were obtained as the 2.5^th^ and 97.5^th^ percentiles of the results of the 1,000 model runs.

## Results

Complete data were available from all 24 participants. The CO_2_ stimuli at concentrations far from the pain threshold were rated according to their strength. Specifically, in “group 1”, 0% of the below-threshold stimuli (25% v/v CO_2_) and 96.7 ± 7.8, 96.7 ± 7.8 and 96.7 ± 11.5% (identical means) of all CO_2_ stimuli well above the pain threshold (75% v/v) were rated as painful after a “no pain”, “stimulus” and “pain” cue, respectively. In “group 2”, this meant that 0% of the below-threshold stimuli and 89.8 ± 16% of the above-threshold stimuli were judged as painful. For all far-from-threshold stimuli, irrespective of the cues and groups, the ratings in this group did not differ significantly from the expectations (p always > 0.05 in all one-sample t-tests against the expected percentages, i.e., 0 or 100% of the 25 or 75% v/v CO_2_ stimuli, respectively).

**Fig 2:**
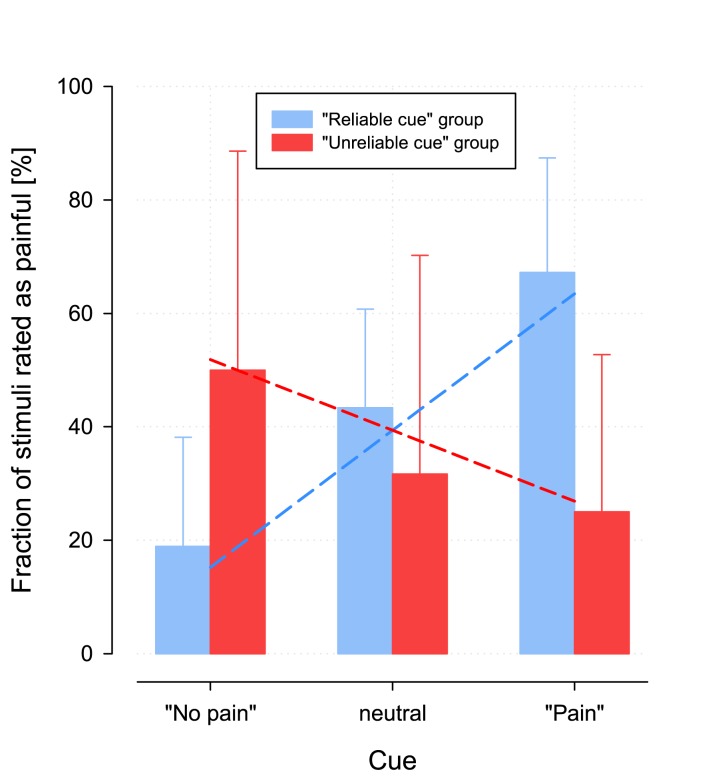
Bar graph of the percentage of stimuli at threshold level that were perceived as painful, separately for each different cue and for the two groups. The **columns and error bars** indicate means and standard deviations. Left (red bars): In group 2, where among the stimuli at pain threshold level, correctly cued, far-from-threshold stimuli were randomly interspersed, a significantly higher percentage of stimuli at pain threshold level were rated as painful after a “pain” cue as compared to stimuli rated as painful after a “neutral” or a “no pain” cue. Right (blue bars, main results): In group 1, where, in contrast, the stimuli at threshold as well as the interspersed far-from-threshold stimuli were randomly and equally often cued as painful, neutral or not painful, s the effect was completely inversed. The group*cue interaction effect was statistically highly significant (Table 2). The **dashed lines** superimposed onto the bar graphs indicate the fits of the linear model used to mathematically describe the relationship between the percentages of stimuli at pain threshold level rated as painful and the three different cues as $PercentRatedAsPain\;=\;39.5\frac{\%}{AU}∙({-0.526}^{2-Group})∙Cue+24.1\%$ (Table 4).

A different pattern of painful perception of the stimuli was observed for the stimuli at pain threshold level. In “group 1”, these stimuli were rated incongruently to the cues (Fig 2). That is, pain threshold CO_2_ stimuli were rated significantly less often as painful when pre-announced as painful than when cued neutrally or as not painful. Vice versa, pain threshold CO_2_ stimuli were rated significantly less often as non-painful when pre-announced as non-painful, than when cued neutrally or as painful (Table 2). This was not observed in the second group. That is, in “group 2”, prior announcement of the expected painfulness of the threshold CO_2_ stimuli resulted in the expected outcome (Fig 2). Stimuli at threshold level were rated significantly more often as painful when cued as such, than when pre-announced as being neutral or as no pain. This group difference among threshold stimuli judged as being either painful or not painful was statistically significant (rm-ANOVA interaction “group” by “cue”: F(2,44) = 24.03, p = 8.85 · 10^-8^; for further statistical details including mean values and standard deviations, see Table 2). According to the value of η^2^, the effect explained more than half of the total variance in the ratings of the stimuli as either painful or not (52.2%). The additional analysis of the certainty of the subjects about their judgement resulted in (i) subjects in the first group were less confident than those in the second group (between-subjects effect “group”: F(1,11) = 0.942, p = 0.011; for further statistical details including mean values and standard deviations, see Table 3) and (ii) all subjects were more confident when rating a stimulus as “not painful” (main effect “pain”: F(1,11) = 4.93, p = 0.048).

**Table 2:** Statistical details of the percentages of stimuli at pain threshold level rated as painful: Top: Means and standard deviations of the pain perception [%] of the CO_2_ stimuli at pain threshold level (“Group 1”: far-from-threshold stimuli randomly announced as “no pain”, “stimulus”, “pain”; “Group 2”: far-from-threshold stimuli correctly announced, i.e. “no pain” and “pain”). **Bottom:** Detailed results of the analyses of variance for repeated measures and the estimates of the respective effect sizes partial eta squared, $\eta_{p}^{2}$. Results are provided for main effects and interactions, with the F-value followed by the number of degrees of freedom in parentheses and the p-value.

Parameter	Results	
Descriptive statistics		
*CO*_*2*_ *stimuli at pain threshold perceived as painful [%]*		
Cue	**“Group 1”**	**“Group 2”**
“No pain”	50.0 ± 38.61	18.89 ± 19.25
Neutral (“stimulus”)	31.67 ± 38.57	43.33 ± 17.41
“Pain”	25.0 ±27.73	67.22 ± 20.19
*Analysis of variance for repeated measures (Effects)*		
	***rm-ANOVA effects***	***Effect size $\eta_{p}^{2}$`****
“Group”	F(1,22) = 0.598, p = 0.448	0.026
“Cue”	F(2,44) = 2.59, p = 0.086	0.105
“Group” by “cue”	F(2,44) = 24.03, **p = 8.85** · **10** ^**-8**^	0.522

*: The value of $\eta_{p}^{2}$ multiplied with 100 gives the percentage of variance explained by the respective factor [47].

**Table 3:** Statistical details of the VAS ratings of the subjects’ certainty about their judgments of the stimuli at pain threshold level as painful or not: Top: Means and standard deviations of the ratings of the subjects’ certainty about their judgment of threshold CO_2_ stimuli as painful or not (“Group 1”: far-from-threshold stimuli randomly announced as “no pain”, “stimulus”, “pain”; “Group 2”: far-from-threshold stimuli correctly announced, i.e. “no pain” and “pain”). **Bottom:** Detailed results of the analyses of variance for repeated measures and the estimates of the respective effect sizes partial eta squared $\eta_{p}^{2}$. Results are provided for main effects and interactions, with the F-value followed by the number of degrees of freedom in parentheses and the p-value.

Parameter		Results	
*Descriptive statistics*			
		**Confidence in the pain judgment of stimuli at threshold [mm VAS]**	
Cue	**Pain judgement**	**“Group 1”:**	**“Group 2”**
“No pain”	Yes (“pain”)	49.1 ± 10.22	64.16 ± 29.23
	No (no pain”)	51.38 ± 6.45	78.7 ± 21.54
Neutral (“stimulus”)	Yes (“pain”)	40.13 ± 20.08	71.11 ± 20.16
	No (no pain”)	50.81 ± 6.45	75.62 ± 16.84
“Pain”	Yes (“pain”)	39.63 ± 23.86	79.5 ± 16.76
	No (no pain”)	51.04 ± 5.48	85.04 ± 19.98
*Analysis of variance for repeated measures (Effects)*			
		***rm-ANOVA effects***	***Effect size $\eta_{p}^{2}$`****
“Group”		F(1,11) = 0.942, **p = 0.011**	0.461
“Cue”		F(2,22) = 0.596, p = 0.574	0.49
“Pain”		F(1,11) = 4.93, **p = 0.048**	0.309
“Group” by “cue”		F(2,22) = 1.776, p = 0.193	0.139
“Group” by “pain”		F(1,11) = 9.2 * 10–5, p = 0.993	0.000
“Cue” by “pain”		F(2,22) = 0.019, p = 0.981	0.002
“Group” by “cue” by “pain”		F(2,22) = 2.218, p = 0.133	0.168

*: The value of $\eta_{p}^{2}$ multiplied with 100 gives the percentage of variance explained by the respective factor [47]

Mathematical modeling, i.e., regression analysis of the data (detailed final model and parameter values are given in Table 4, plots of the fits are shown in Fig 2), by placing the number of stimuli at pain threshold level rated as painful in a linear relationship with their respective cues (numerically re-defined as -1, 0 or +1 for “no pain”, neutral or “pain” cues, respectively), provided the statistical basis for a more precise interpretation of the differences detected by means of ANOVA. The first result of this regression analysis was a significant increase or decrease in the number of physically identical, nociceptive stimuli at threshold level, judged as painful when announced as no pain or as painful, respectively, when compared with the neutrally announced stimuli. This significant change was reflected in the positive slope with a confidence interval not including zero (Table 4).

**Table 4:** Parameters of the final regression model. Specifically, the percentages of stimuli at pain threshold level rated as painful were associated with the three different cues using a linear model of $PercentRatedAsPain=Slope\cdot \theta_{1}^{2-Group}\cdot \theta_{2}^{Sex}\cdot Cue+Y\_Intersection\cdot \theta_{3}^{2-Group}\cdot \theta_{4}^{Sex}$, where *Cue* = [–1,0,1] for “no pain”, “stimulus or neutral cue, and “pain”, respectively; *Group* = [1,2]; and *Sex* = [0,1] for women and men, respectively. The fits are plotted in Fig 2. The final model was the result of goodness-of-fit statistics. Only parameters that provided a statistically significant improvement when free to be estimated remained part of the final model, which therefore, in its short form reads as $PercentRatedAsPain=39.5\frac{\%}{AU}\cdot \left(-0.526^{2-Group}\right)\cdot Cue+24.1\%$.

Parameter	Population central value (% SEE)	95% CI*
*Slope* [%/AU]	39.5 (4.8)	30.7 .. 48.9
*Θ*_*1*_	-0.526 (0.6)	-0.945 .. -0.266
*Θ*_*2*_	1 (fixed)	-^#^
*Y-Intersection* [%]	24.1 (4.6)	15.7 .. 32.3
*Θ*_*3*_	1 (fixed)	-^#^
*Θ*_*4*_	1 (fixed)	-^#^

%SEE = percent standard error of parameter estimate,

*95% confidence interval of the parameter obtained as the 2.5^th^ and 97.5^th^ percentiles of the results of 1000 model runs using Bootstrap resampling with NONMEM.

AU: Arbitrary unit used for the above-mentioned numerical re-definition of the cues.

^#^: Not estimated because the parameter was not part of the final model as it had not provided a significant improvement of fit during the model building (fitting) process.

The second and most important result of the linear regression analysis was that the discord between cues and pain ratings for stimuli at pain threshold level was actually reversed to an inverse relationship and not just abolished when the cues were presented randomly with equal chance to announce the three categories of nociceptive stimuli that made them unreliable. Thus, in “group 1”, the slope of the linear relationship between number of painful-rated stimuli and the respective cue was multiplied with a negative factor (Δ_-2LL_: -20.078, p < 0.0001) of -0.526, with a 95% confidence interval of estimate, obtained from 1,000 Bootstrap runs, not including zero [-0.945 .. -0.266] (Table 4). The negative nature of the linear relationship confirms the reversal of the effects of the cues rather than their mere extinction.

The third result was that the difference in experimental conditions between the two groups, affected solely the directed cues while the neutral cues remained unaffected. This was concluded from the rejection of two different y-intercepts from the final model, based on goodness-of-fit statistics (Δ_-2LL_: -1.386, p > 0.05). Finally, modeling established that the inclusion of both sexes in only one of the groups had no major effect on the findings, since the factor “sex” was rejected from the final model (Δ_-2LL_: -0.146, p > 0.05).

## Discussion

The same nociceptive stimulus, repetitively applied but pre-announced randomly as painful, non-painful or neutral, was rated as the opposite of the announcement when the subjects could not rely on the pre-announcement. Thus, the present results show that spontaneous activation of associations that are incongruent with a given message, as previously reported in other contexts than pain [21], can also be observed for the perception of pain. In the present main experiment, in group 1, we created an experimental context in which the subjects’ judgment of the painfulness of a stimulus was opposite to the suggestion implied by the presented cues, i.e., stimuli announced as painful were judged as non-painful and vice versa. This is novel, since previous experiments, while having shown that uncertainty of predictive information may increase pain intensity [53] and that the ability to form accurate expectations decreases the threat [54], a significant effect in the opposite to the cued direction, i.e., a reversal of the subject’s assessment of the painfulness of a stimulus, has never been described. The present pattern of results differs substantially from previous observations under altered certainty conditions [18,19] and may be interpreted as an implicit cognitive strategy for coping with counterfactual information [55].

In an attempt to obtain a basis for the interpretation of these observations, a second study group was subsequently enrolled. However, due to the unbalanced experimental design of this study as a whole, a head-to-head comparison between the effects in the two groups is restricted as they differed with respect to five main characteristics: Firstly, there was a sex difference, with both sexes present in the first group but only women in the second group. Secondly, the groups differed with respect to the applied cues given for the far from pain threshold stimuli, which were random and often incorrect with respect to the announced pain in the first group but always correct in the second group. Thirdly, the groups differed with respect to the number of administered stimuli, with more far-from-threshold stimuli in the first group than in the second group, but more pain threshold stimuli in the second group. Fourthly, the duration of the experiment in the second group was longer than that in the first group. Fifthly, subjects in group 2 were on average 2.6 years older than the subjects in group 1.

An influence of the first main difference, the sex imbalance, could be excluded based on the results of the mathematical modeling. Specifically, this could be unambiguously concluded from the rejection of the factor “sex” from the final linear model because its inclusion improved the goodness of fit only non-significantly, as judged by the likelihood ratio test (Δ_-2LL_: -0.146, p > 0.05). Applying the principle of parsimony (see also http://en.wikipedia.org/wiki/Occam%27s_razor), “sex” was therefore not a part of the final linear regression model.

An influence of the second main difference, the correctness of the cues given for the far from pain threshold stimuli, seems to offer a suitable path toward interpretation of the present observations. However, as we only assessed how certain subjects rated the stimulus, the mechanism underlying the present observations can only be deduced via interpretation of previous findings. Of particular utility for this purpose is an experiment in which the presence of an untrustworthy face conveyed faster activation of incongruent than congruent associations [21]. This was interpreted as being due to an environment of distrust [21]. Furthermore, even when the distrust was unrelated to the message, the cognitive system reacted to distrust by automatically inducing the consideration of incongruent associations. This is remarkably similar to the present observations made in group 1, in which subjects were confronted with incongruent cue-stimulus associations. Hence, distrust, or alternatively unreliability of the cues, seems to be a likely explanation. Indeed, distrust has been suggested to lead to spontaneous consideration of alternatives [56]. In the present study, despite the fact that subjects in the first group rated the stimuli as painful almost equally as often as in the second group, the subjects of the first group rated the effects of the stimuli contrary to the indications of the specific cues to a statistically significant extent.

A unique feature of our experimental design is, that the subjects in the first group were confronted with randomly, often incorrectly assigned cues, prior to the far-from-threshold stimuli, which were easily recognizable from their quality. Subjects were aware of false announcements as correct ratings of painfulness despite misleading cues shows. This condition of clear discrepancy between cue and stimulus was necessary to generate a context of questioning the cues, as we expected that the frequent violation of the subjects´ expectations may have created an untrustworthy environment, triggering suspicion as a central cognitive component of distrust [57]. In this environment, the suggestive effects of the cues were not only abolished but reversed to their opposites. The lower confidence of the subjects of group 1 in their judgment about whether or not a stimulus at pain threshold level was painful points into the same direction, however, the query of the certainty of the subjects’ judgment cannot fully replace the lack of the query about the trust in the given cues. We expected to learn from this paradigm how subjects deal with clear false information about pending pain experience as previously shown in another context involving invalid messages associated with faces eliciting trust or distrust [21]. Even if stimuli were falsely announced by e.g. visual cues [4,58] or varying expectation intervals [59], differences in applied stimulus intensities were so small (e.g. 1°C and 2°C, respectively [4,59]) that they were probably not noticed. This is also supported by the subjects’ rating according to elicited expectation. This component of distrust clearly distinguishes the present results from previous reports, which, for instance, simply increased the probability of rating stimuli at pain threshold level as painful by connecting the stimuli suggestively with potential harm [9]. Subjects in our second study group, confronted with congruent cue-stimulus associations, on the other hand, were given the correct cues for the quality of subsequent stimuli far from the pain threshold. This differed from the design of previous assessments where no such clear hints were provided (Table 1). Thus, noxious stimuli clearly above pain threshold were pre-announced as “painful” while clearly non-noxious stimuli were cued as “non-painful”. This created a reliable context for all presented cues, which probably enhanced their suggestive effect, with the result that subjects were unsuspicious and trusted the cues, which previously had required stronger suggestions, e.g. associated with a threat. Indeed, in another preliminary experiment, which omitted the far-from-threshold stimuli (data not shown), the cues had no effect.

The supposition that the context of the experiment in the first group resembled unreliability, or distrust, provides a clinical interpretation. Thus, in relation to patients [60], trust has been conceptualized as a set of beliefs that the therapist will behave in a certain way [61], which usually comprises several characteristics of the therapist, such as competence, compassion, confidentiality or reliability [62]. Identification by patients of false, euphemistic information about medical interventions may severely disrupt the patients’ trust in the physicians and results in potentially exaggerated experience of pain [63]. The present experiment, in some ways, reflects such a situation, suggesting that distrusted or unreliable cues may not only be ignored but in the extreme, will be discounted, with the consequence that the diametrically opposed alternative will be considered as more likely.

The third difference between the two groups was the number of stimuli administered. This, retrospectively, was an unfortunate design, posing problems in interpretation as the second group does not represent an adequate control. In the second group, less far-from-threshold stimuli and more pain threshold stimuli were administered than in the first group. The first difference was partly accounted for by the absence of far-from-threshold stimuli announced neutrally in group 2. However, the smaller number of these stimuli might have jeopardized the creation of a true, reliable context and the subject might simply have followed the cues without having learned about their reliability from their consistency with the far-from-threshold stimuli. In this case, the experiment in the second group would simply have reproduced previous observations in which dirigible pain perception was observed when announcements correctly predicted the painfulness of imminent nociceptive stimuli (Table 1). This challenges the contrast between the contextual reliability of the cues for the two different study groups. However, it does not challenge the observation in the first group, in which subjects inversely rated the threshold stimuli in relation to the given cues. The interpretation, thus, remains valid that the condition employed in this group resembles a context of unreliability, which has been observed previously to produce similar behavior in other experimental settings than used by us. Fourthly, although group 2 participated in a longer experiment as group 1, the consequence of this remains unclear. Due to tiredness, the subjects might have been more willing to simply follow the cue rather than judge the painfulness of the stimulus. However, the total duration was not excessively long in experimental human pain research. Finally, the small age difference seems unlikely to have influenced the results to a major extent. The ages of 23.9 and 26.5 years are usually regarded as belonging to the same age group; for example, the age-stratified reference data for the quantitative sensory testing battery of the German Research Network on Neuropathic Pain comprise an age group of 20–30 years [64].

The present study demonstrated the induction of associations incongruent with a given message in the perception of pain. Random and often incorrect announcement of stimuli clearly below or above pain threshold caused the subjects to judge the painfulness of stimuli at pain threshold level as the opposite of the suggestion implied by the presented cues. As a possible interpretation, the experiment was set in an untrustworthy or unreliable context, probably placing the subject in a suspicious, distrustful state, and the cues triggered pain perceptions in the opposite direction, inducing a tendency for the subjects to prefer the diametrically opposed alternative to the cues. This has been shown in other contexts and the present results indicate, for the first time, that the same behavior can also be observed in a pain context. The results emphasize the particularly complex psychophysics occurring at the pain threshold, which are obviously subject to many different modulatory effects, but may play a significant role in the successful treatment of pain in a clinical setting.

## Supporting Information

S1 Data The file S1_Data.txt contains the source data as a tab delimited ASCII text file providing the data, namely the percent of threshold stimuli rates as painful, as a matrix of subjects (rows) and variables (columns) with self-explaining column headers. (TXT)
